# Understanding existing research culture and capacity in a local authority (LA) and voluntary, community, faith and social enterprise(VCFSE) in one area of the South West, United Kingdom: a qualitative baseline study

**DOI:** 10.1186/s12961-026-01498-x

**Published:** 2026-08-10

**Authors:** Jack Brazier, Jessica Horner, Jemimah Allwright, Owen Bell, Oliver Clabburn, Emily Dodd, Emily Hutt, Alexandria Hewitt, Adam Lane, Frederick Marais, Alice Munro, Jane Powell, Ria Saha, Brittney Strange, Lou Woolway, Hamed Zandian, Alison Bell

**Affiliations:** 1 Health Determinants Research Collaboration, Public Health, Somerset Council, County Hall, The Crescent, Taunton, United Kingdom; 2 Centre for Public Health and Wellbeing, University of the West of England, Frenchay Campus, Coldharbour Lane, Bristol, United Kingdom; 3 Spark Somerset, Bowdens Farm, Hambridge, Langport, United Kingdom; 4 Public Health Wales, 2 Capital Quarter, Tyndall Street, Cardiff, United Kingdom; 5 National Health Service, Somerset Integrated Care Board, Wynford House, Lufton Way, Yeovil, United Kingdom

**Keywords:** Research culture, Research capacity, Community participation, Local authority, Health determinants, Health inequalities, Research systems

## Abstract

**Background:**

Local authorities play a pivotal role in addressing health inequalities and inequities by tackling their underlying social determinants – the fundamental building blocks of health. Research is therefore vital to ensure that decisions are evidence-based and tailored to population needs. Health Determinants Research Collaborations offer an opportunity to build research capacity and culture within local authorities and voluntary, community, faith and social enterprise (VCFSE) settings; however, an understanding of existing research practices and culture is required. Our study aimed to gather a baseline understanding of this within these settings in Somerset, United Kingdom.

**Methods:**

We utilized a multiple-methods qualitative study design. A total of 46 local authority employees took part in focus groups (*n* = 12) and in semistructured interviews (*n* = 1). An additional 18 participants from local VCFSE organizations took part in focus groups (*n* = 1), interviews (*n* = 8) and through completion of an online qualitative survey (*n* = 4).

**Findings:**

Using framework analysis, we identified four themes across the local authority and VCFSE datasets: (i) perceptions and value of research and evaluation; (ii) community participation in research and evaluation; (iii) utilization of data and evidence; and (iv) research and evaluation training needs.

We found clear differences between the local authority and VCFSE sectors in how they define, use and resource research. Participants in both sectors described research as highly valued, but many equated it primarily with data collection and application, rather than with the delivery of rigorous academic research studies.

Participants emphasised the need to integrate lived‑experience data more effectively into decision‑making. They suggested this integration is achievable through stronger community involvement structures and closer collaboration with the VCFSE sector.

Although the local authority often draws on quantitative data to inform decision‑making, there was a recognized need for making greater use of qualitative data. Additionally, participants reported that challenges with data usability and data‑sharing processes and the lack of integrated datasets limit their ability to fully use available evidence.

Finally, participants identified a broad range of research and evaluation training needs, particularly training that supports participatory approaches, mixed‑methods data collection and analysis, and evidence interpretation and application.

**Conclusions:**

Our findings demonstrate the complexities of building research capacity and culture within local authority and VCFSE settings and highlight significant opportunities for collaboration and influence on organizational and system decisions. The identification of levers and barriers to research within these settings offers novel insights for future funders, policy-makers, researchers and practitioners seeking to build research capabilities and culture.

**Supplementary Information:**

The online version contains Supplementary Material available at https://doi.org/10.1186/s12961-026-01498-x.

## Background

**Fig. 1:**
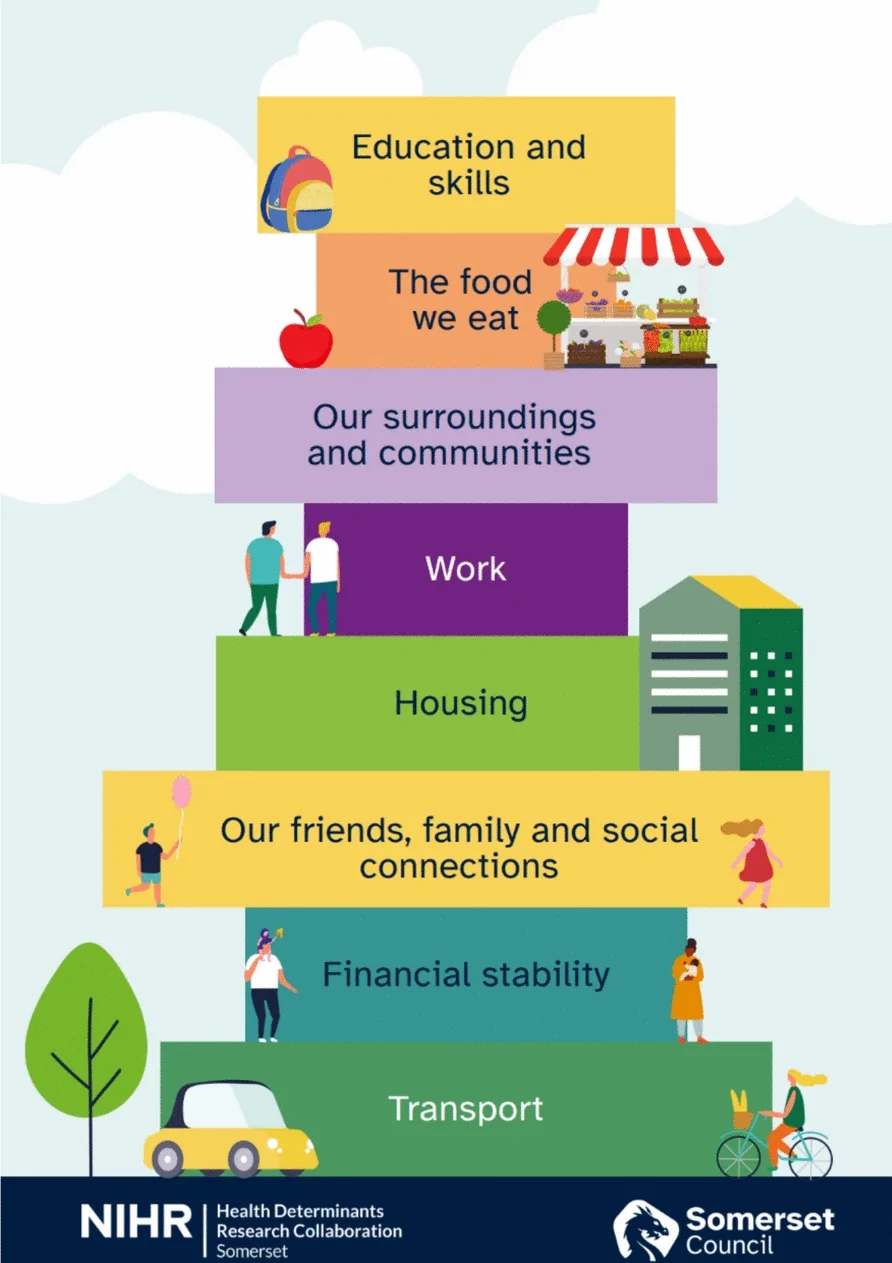
The building blocks of health (adapted from The Health Foundation) [4, 10]

Despite considerable improvements in health trends in England over time, health inequalities and inequities persist [1]. For example, those living within the least deprived deciles of the country live in good health, approximately 20 years longer than those living in the most deprived communities [1]. Health is one component of the Indices of Multiple Deprivation. The wider determinants of health, such as education, food security, employment, housing, transport and social connectedness, are fundamentally interconnected with deprivation. Deficits in these areas increase the likelihood of poorer health outcomes, underscoring their role as the essential building blocks of health (Fig. 1) [1–4].

Recent investment in local authority research infrastructure across the United Kingdom demonstrates the importance of building sustainable research cultures and capacity to reduce health inequalities [5]. Specifically, greater national-level investment in research culture should be channelled into: (i) sourcing funding and leadership; (ii) establishing internal research processes, systems and training; and (iii) working more closely and in partnership with communities and Voluntary, Community, Faith and Social Enterprise (VCFSE) organizations [6–8]. In response, the National Institute for Health and Care Research (NIHR) funded 30 Health Determinants Research Collaborations (HDRCs) across England, Scotland and Wales to embed research capability within local authority systems. Local authorities are responsible for providing statutory, essential services, such as Adult Social Care, Children and Families services, Economic Development, Housing, Planning, Public Health and Transport [9]. These services all have strong links and responsibilities concerning the wider social determinants or building blocks of health (Fig. 1).

These are sectors that directly impact population health but have limited histories of conducting or applying research despite being mandated to engage in research within national policy and legal frameworks. By upskilling the local authority workforce in research, evaluation, community participation and evidence use, HDRCs support these services to integrate research evidence into routine decision-making, reducing health inequalities by developing effective and equitable policies and services.

To reduce health inequalities and improve health outcomes, the effective use of research is essential to ensure that local authority decision-making, including policies, services, strategies and interventions, are designed with the best available evidence and tailored to local population needs and demographics [11–13]. Local authorities face numerous challenges due to changing political contexts and are often required to work within restricted financial capacity and human resource [14–16]. Such challenges have similarly been highlighted as major barriers in developing and sustaining research cultures within local authorities, thus limiting capacity to sustainably integrate research into organizational structure and culture [7, 8].

One way to overcome these challenges is the embedded researcher model. Experienced researchers are either seconded or employed by local authorities in fixed-term posts to promote the use of research and evaluation and build pathways between academia and practice [17–19]. Whilst the embedded researcher model demonstrates signs of increased research activity and engagement, literature has similarly highlighted limitations in the approach [17, 19, 20]. Namely, embedded researchers’ ability to navigate complexity in unfamiliar settings, the need for research to be conducted and published at speed, acting as a single resource within a large organization, and a lack of clear aims for embedded researchers to work towards, are well documented challenges within embedded researcher models in local authorities [17, 19, 20]. Moreover, such approaches do not provide adequate capacity for meaningful community participation in research, with evidence drawn exclusively from studies exploring embedded researcher models within Public Health departments only. Therefore, little is known about developing and establishing research cultures throughout local authorities as whole organizations. For sustainable and effective research cultures to thrive within local authorities, substantial and longer-term investment, which spans the services most connected to building blocks of health, is required [6, 7].

### Context

In the second wave of NIHR funding for HDRCs, Somerset Council, in partnership with Spark Somerset, University of the West of England (UWE Bristol) and the Institute of Health Equity at University College of London, was awarded 5 million pounds sterling to develop a HDRC between 2024 and 2028 [21]. Somerset HDRC seeks to build research and evaluation capacity through training programmes, support functions, infrastructure development, funding generation and strategic partnerships with academic and community organizations. Moreover, Somerset HDRC is committed to building a research culture which embeds the lived experience of local communities into all research and decision-making through partnerships with the VCFSE sector and local community. Our HDRC model and business plan includes the local VCFSE sector as a key partner in building research and evaluation capacity within the Council, recognizing their role in reaching communities and supporting meaningful local research and evaluation.

Similar to many local authorities in the United Kingdom, Somerset Council has recently undergone major structural changes, including transitioning to a unitary authority, implementing a restructure programme and developing a strategic transformation plan [22, 23]. These shifts not only create financial and resourcing pressures but also offer an opportunity to embed a stronger research culture within the Council’s future decision‑making. This makes the HDRC both timely and highly relevant for the local authority and the communities it serves. To achieve this, there is a need for context‑specific insights into organizational research capacity and requirements [24]. Bell et al. [24] outline methods used by HDRCs to assess research culture and capacity, although much of the existing evidence is based on survey data. Whilst valuable, surveys offer limited contextual depth compared with qualitative approaches such as interviews and focus groups [25]. To address this gap and enhance the potential impact of this new research award, our study uses qualitative methods to provide an in‑depth understanding of research culture and capacity, training and support needs, strengths, interests, and priorities within the local authority and local VCFSE sector.

To address our research aim, the following research objectives were set:

i. To understand the perceptions, value and use of research within a South West local authority and amongst VCFSE stakeholders.
ii. To explore how lived experience data are currently utilized to inform decision-making within the local authority and VCFSE sector.
iii. To capture the research training, infrastructure and support needs within the local authority and VCFSE organizations.
iv. To identify opportunities for strategic research collaboration between the local authority and VCFSE sector.

## Methods

Our study reports Somerset HDRC’s baseline assessment of research culture and capacity within the local authority and local VCFSE organizations, building on the methodological summary presented by Bell and colleagues [24]. The Consolidated Criteria for Reporting Qualitative Research (COREQ) checklist has been utilized to provide a comprehensive and rigorous description of our research design, participants and analysis in this paper [26] (Supplementary Material 1).

### Study design

An exploratory multiple-method qualitative study design was adopted, incorporating semistructured interviews, focus groups and a qualitative survey [27]. This design enabled triangulation of perspectives across data collection methods and organizational contexts. Interviews and focus groups provided in-depth insights, whilst the qualitative survey facilitated inclusive and broader participation across services and organizations.

### Recruitment

Purposive sampling was adopted to recruit participants from both local authority and VCFSE sectors, by either approaching all Somerset Council services, which held responsibility for the building blocks of health (Fig. 1), or via circulating direct emails to VCFSE organizations known to Spark Somerset, the county’s voluntary infrastructure organization. Upon receiving an expression of interest, participants were provided with a participant information sheet and consent form, before being invited to a focus group or interview. Allocation to interviews or focus groups was based on participant preference and availability, alongside logistical considerations to maximize participation. Participants who declined these options were subsequently offered to take part via qualitative survey. It was essential to have a range of representation across different council functions and organizations, therefore recruitment was stopped at the point of data saturation and once a representative sample was obtained.

### Data collection

#### Local authority setting

Interviews and focus groups were conducted by 11 of the 14 researchers involved in the study who possessed varied experience in qualitative health research. Those with a greater level of experience and knowledge in qualitative research methods (A.M., B.S., E.D., F.M., J.B. and O.C.) acted as facilitators of interviews, whereby those with less experience supported data collection as co-facilitators, thereby facilitating upskilling within the internal HDRC team. Co-facilitators supported note-taking and logistics, whilst facilitators led discussions. All team members followed a shared topic guide and internal guidance to ensure consistency. Data collection was carried out between August 2024 and January 2025. Both face-to-face and online appointments were offered to participants to enhance accessibility. The interview guide, focus group prompts and survey instrument are provided as Supplementary Material (Supplementary Material 2).

#### VCFSE sector setting

Data were collected with stakeholders from VCFSE organizations according to participant preference through focus groups, interviews and online qualitative survey, comprised primarily of open-ended questions designed to capture participants’ perspectives, with a small number of closed-ended questions used for contextual information. Where possible, focus groups and interviews were undertaken in person, located at either the participants’ workplace or community space, and led by a topic guide that was adapted to reflect the specific context of VCFSE organizations, with minor adjustments to language and examples to ensure relevance whilst maintaining consistency across data collection. Online formats were also provided to enhance accessibility and engagement. For organizations that did not wish to participate in focus groups or interviews, a qualitative survey was administered via Microsoft Forms.

Ethical approval was obtained from the Research Ethics Committee at the University of the West of England (UWE Bristol) (REC reference: CHSS.24.06.220).

### Data analysis

Focus groups and interviews were audio-recorded using dictaphones for in-person sessions and Microsoft Teams for online data collection. Additionally, co-facilitators documented reflective notes on key points of discussion, future research priorities and requests for HDRC support. Verbatim transcriptions were created by an external transcription service and members of the team. All anonymized transcripts were securely stored on a SharePoint with access restricted to the research team. Transcripts were not returned to participants for comment or correction owing to competing demands within the local authority and VCFSE settings. Whilst initial coding was inductive, the analytical framework was iteratively refined in relation to the study objectives to support structured comparison across datasets.

Framework analysis was utilized to explore and compare data between services and settings. This approach enabled the utilization of individual team members’ knowledge and expertise for collective analysis. Any discrepancies in coding were discussed within pairs and, where required, resolved through team discussion to ensure consistency in interpretation. Analysis followed guidance published by Gale et al. [28] and is outlined in Table 1.

**Table 1:** Stages of framework analysis [28]

Stage	Authors involved	Process undertaken
1. Transcription and cleaning	J.H./O.C./external transcription service	Identification and commissioning of external transcription service
2. Familiarization	A.H./A.M./B.S./E.D./E.H./F.M./H.Z./J.B./J.H./O.B./O.C./R.S.	Familiarization with the data and initial ideas were formed through data collection. Formal analyses began with the review of four transcripts (local authority focus groups: *n* = 2; VCFSE interviews *n* = 2), whereby initial codes were developed. This was achieved through ensuring all transcripts were anonymized and formatted correctly. Preliminary thoughts and notes were collated to inform stage three of analysis. This phase was repeated by all authors with their assigned transcripts in stages five and six
3. Coding	A.M./F.M./J.B.	An inductive approach to coding was undertaken in line with recommendations to ensure rigour [28]. Authors held a specific focus on substantive (e.g. behaviours and structures) and value-based (e.g. beliefs and desires) codes in line with the study aims [28]. Authors carried out coding in individual Microsoft Excel files
4. Developing an analytical framework	A.M./F.M./J.B.	Codes were then compared and contrasted. Overall, there was consistency and similarity amongst the majority of codes. The most significant differences were identified between local authority (LA) and VCFSE transcripts. The four individual coding frameworks were amalgamated into one analytical framework and then reviewed. All authors then met to discuss the analysis process and explain the framework
5. Applying the analytical framework	A.H./A.L./A.M./B.S./E.D./E.H./F.M./H.Z./J.B./J.H./O.B./O.C./R.S.	Authors with greater experience in qualitative data analysis were paired with authors holding less knowledge of analysis techniques. This process supported both internal skill development within the HDRC team, and intercoder reliability for the study. Each pair was assigned a minimum of one transcript and given a template of the analytical framework in Microsoft Excel. After each pair had analysed one transcript, the team met to discuss any iterations needed for the remaining transcripts. Subsequently, four additional codes were included, each supported by sub-codes
6. Charting data into the framework	A.H./A.L./A.M./B.S./E.D./E.H./F.M./H.Z./J.B./J.H./O.B./O.C./R.S.	Individual quotes were extracted against the codes outlined in the framework by the authors. Quotes were then categorized and organized into themes and subthemes, with transcript references, participant identification numbers and transcript line numbers. Themes and subthemes were then charted to create summaries
7. Interpreting the data	A.H./A.M./B.S./E.D./E.H./F.M./H.Z./J.B./J.H./O.B./O.C./R.S.	All analysts were encouraged to note key points of interest and strategic opportunities throughout their analyses. Notes were captured within pairs’ analytical frameworks and later consolidated. The writing up of findings was firstly presented for comment to the HDRC team, followed by strategic stakeholders involved in the study

A sample of the analytical framework utilized to guide analysis for the study, including coding structure and definitions (Supplementary Material 3), is provided in the Supplementary Materials.

### Reflexivity

Given the crossover of affiliations between many members of the research team and participants, it is important to consider reflexivity within this study. J.B. led the recruitment of participants to the study and held no prior relationship with participants except for those working within the Public Health service. Whilst Public Health staff were purposively recruited, this was conducted via a webinar describing the objectives, purpose and structure of the HDRC, and for this study, an expression-of-interest QR code was made available to attendees, thereby adopting a less direct approach to recruitment to lower any risk of influencing participants’ decision to take part. To provide participants with an understanding of the researchers’ positionality, introductory emails and participants information sheets outlined the HDRC as an externally funded Somerset Council function with overarching, funder-determined objectives to meet. At the start of each focus group or interview, facilitators introduced themselves and stated their organizational affiliation to outline their role within the HDRC team.

## Findings

In total, 64 participants took part in the study. Within the local authority setting, data were collected from 46 participants across 12 focus groups and one individual interview, with group-based participation reflecting service-level engagement within the local authority. Participants represented teams across Adult Social Care (*n* = 2), Children and Families (*n* = 3), Economic Development (*n* = 2), Education (*n* = 1), Housing (*n* = 5), Local Community Networks (*n* = 10), Planning (*n* = 5), Public Health (*n* = 10), Revenues and Benefits (*n* = 2), and Transport (*n* = 6). Additionally, 18 participants representing the VCFSE sector took part via semistructured interviews (*n* = 8), qualitative surveys (*n* = 4) and focus groups (*n* = 1), representing small, medium and large organizations across the county.

Our framework analysis identified four primary themes: (1) perceptions and value of research and evaluation; (2) community participation in research and evaluation; (3) utilization of data and evidence; and (4) research and evaluation training needs. All themes were present across data collected within local authority and VCFSE settings and were supported by descriptions illustrating the overall meaning of each theme (Table 2).

**Table 2:** Theme and descriptions

Theme name	Theme descriptions	
	Local authority setting	VCFSE setting
Perceptions and value of research and evaluation	This theme suggests research is perceived as the collection and application of data or evidence, rather than utilizing published, so-called academic research. Typically, research is used for service, strategy and project development, ensuring accountability and justification for decision-making. However, time, capacity, competing demands, a lack of supporting infrastructure and local authorities’ ways of working hinder staffs’ ability to engage with research	This theme suggests conflicting perceptions of research amongst VCFSE participants. In some instances, research was perceived to be useful for evidencing impact of projects, annual reporting, funding generation and identifying community need. However, data suggest engaging in research is a time-consuming process, can be subject to manipulation and is often perceived as an activity for those in academia or medicine. Community engagement and conversations were regularly conducted to capture community needs and evaluate projects, yet are not identified as research, reflecting how differing terminologies concerning research are adopted between sectors
Community participation in research and evaluation	Data suggest both a desire and need to better integrate the voice of communities into strategy, policy and service design within the local authority. It is suggested that generally a top-down (i.e. feedback surveys and consultations) approach to community participation exists, yet there is ambition to shift to a bottom-up approach which focuses on community engagement. To overcome barriers to community participation, building meaningful relationships with communities and clear pathways to influence policy and practice are essential	Similarly to the local authority, there is interest to better support meaningful community participation and engagement for research, and a need to move away from VCFSE organizations being perceived as a recruitment mechanism. Participants suggest the sector can offer opportunities to build more equitable research partnerships with communities; however, the ask of researchers upon both community sector organizations and communities must be clearly articulated
Utilization of data and evidence	Routinely collected quantitative data (often gathered as key performance indicators as per statutory requirements) are utilized more frequently than qualitative evidence. Typically, national and internal (local) data sources, as opposed to published research works, are used within day-to-day roles and decision-making. However, the usability of such data, difficulties in accessing and sharing data, and limitations in the relevance of national datasets at a local level, often result in the underutilization of evidence in decision-making	Within the community sector, lived experience is recognized as an important and commonly utilized source of data, typically collected via case studies, testimonials, feedback surveys and community conversations. Furthermore, whilst local and national secondary datasets are used for project design, the ability to share data between organizations and sectors was deemed a considerable barrier to greater use of such data; a shared challenge between sectors
Research and evaluation training needs	Training needs and interests within the local authority setting were categorized into three areas: (i) data collection and analysis; (ii) evaluation; and (iii) fundamental research principles. These broad training areas included specific topics such as designing robust research questions, qualitative and quantitative study design and analysis techniques, and co-production. Moreover, training in evaluating policy, services and projects was sought after, with specific interest in both realist and economic evaluation. Lastly, training should embed fundamental research principles, such as data quality, critical appraisal, research ethics and knowledge mobilization	Contrasting views on the benefits of research training were evident within this theme; however, the value of light-touch training outlining the principles and broader concepts of research, such as identifying data sources, removing biases, or data validity or reliability, was highlighted. Moreover, findings suggest that training in novel or creative approaches to collecting lived experience evidence is required. Thus, enabling community sector staff to confidently communicate and disseminate research opportunities may support intrasectoral (system) approaches to undertaking research

### Perceptions and value of research and evaluation

Our data suggest that the term “research” within a local authority setting may differ from definitions or perceptions of research held within sectors that have traditionally undertaken or utilized research, such as academia or medicine. Research was determined as the collection and application of data. Local authority staff acknowledged that data may not be collected or analysed using rigorous or systematic processes common within academia. However, routinely collected data gathered through statutory processes are often used by local authority services to inform, develop, and refine policies, projects and strategies. This demonstrates the perceived value of research in a specific local authority context.

It’s something we don’t do very often in that pure sense, because studying something in-depth isn’t something we do a lot of. So, often we’re not working in an academic note, and it tends to be that from the planning perspective… a lot of it is about monitoring, understanding what indicators are, [and] gathering information, not necessarily researching. – Local authority (Planning)

We’re thinking about data, data intelligence, we’re thinking about best practice, and evidence, and it’s kind of all those kind of different aspects of research. And it’s kind of where in the process of this life cycle of research [are we]? What do we do well and where don’t we do so well? – Local authority (Public Health)

Within the VCFSE sector, research was similarly defined by terms such as “finding out information”, “identifying issues” or “benchmarking”. Often highlighted were the application and use of data for evidencing impact and funding applications. In contrast to this, research was perceived as a process that is associated with academic institutions as opposed to the VCFSE sector.

I think a lot of small organisations would see it as something academic and scary and for books and statistics. – VCFSE organization

People are Doctors of research. People aren’t doctors of evaluations. I think research has got a very academic reputation. – VCFSE organization

Time and capacity were presented as significant barriers to undertaking and engaging with research across both local authority and VCFSE sectors, whereby the demands of delivering and sustaining services take priority. Specifically, within the local authority setting, participants suggest that the timelines of designing, delivering and disseminating research projects do not align with the needs with delivering statutory services in an ever-changing sociopolitical environment. Consequently, to enable local authority staff to undertake and utilize research, supporting infrastructure and tools, such as evaluation frameworks or integrated datasets, is required.

Main barrier I think is resource. We’ve got a great data collection team, but there’s not very many of them [and] it takes up a lot of their time. – Local authority (Transport)

Moreover, to support research alongside the VCFSE sector, requests for collaboration must clearly outline how a project aligns with the organizations’ aims and ensure that the research tasks are embedded into the delivery of projects and do not detract from project delivery. However, the most significant enabler of research across both settings is suggested to be improved intra- and intersectoral collaboration, whereby shared priorities are identified and organizational barriers to partnerships working are actively dismantled.

The two biggest things – capacity, and I guess – interest, as well maybe... Well, you know, I guess putting it more politely, is seeing the relevance to your organisation and making the time to do it. – VCFSE organization

I think our procurement process, and it’s going to get worse with the new legislation, it’s quite a competitive process and what it then does is put all of our partners in competition with each other naturally. Whereas what we’re trying to do is adopt/foster a collaborative space in Somerset. – Local authority (Children’s Services)

### Community participation in research and evaluation

The need to better integrate community voices into local authority service, strategy, policy and project design is demonstrated within our data. Participants acknowledged that existing practice predominantly employs a top-down approach. Such an approach relied upon feedback surveys and consultations of predetermined Council plans. This creates risk of not capturing the views of those who are more likely to suffer from health inequality and inequity.

I feel like we often do that kind of top down [approach], ‘we think we should look at this’, whereas it would be good to be able to go to the communities that we’re working with and say ‘what matters to you? What do you think we should look at?’, and following their lead rather than our lead. – Local authority (Public Health)

In an ideal world, we would have lots of engagement and consultation with members of the public, but again, it’s constrained by time and resource. At the moment, we’ve got a certain amount of time to try and get the plan adopted, but potentially, with the new government, that timeframe being pulled even shorter, which then will have a direct impact because those consultation and engagement activities are quite time intensive – Local authority (Planning)

However, there was evidence of co-designed plans, which integrated lived experience into the planning, writing and dissemination of strategies. Our data suggest a unanimous desire to shift local authority culture towards a bottom-up approach which embeds community participation into standard practice within Council processes. Historically this has not enabled meaningful community involvement or collaboration. Yet, to do so, meaningful relationships and trust must be built with communities. Foundations must be established whereby community members are perceived as equitable partners in projects and demonstrate how the local authority will integrate lived experience into service, policy and project design.

Quite often we get short pots of money confirmed at the very last minute with a really short turnaround, [the] Household Support Fund is a prime example of that. [We] got notified at the end of September to step up in the 1st October, and it was just like, ‘oh my God’, how on earth? Then you’ve got to jump through all the hoops in terms of like procurement and finance here as well. So, there is something in terms of our bureaucratic structures [that] actually do not enable that co-production or the collaboration. – Local authority (Children’s Services)

It’s having that luxury of time to be able to do it in a meaningful way, and not at the detriment that it then becomes a tick box. – Local authority (Children’s Services)

VCFSE participants indicated that their organizations are frequently approached to undertake consultations, or to recruit community members to research studies. However, a lack of clarity regarding the ask of VCFSE organizations, ambiguity regarding how a project may benefit communities and limited involvement in the design of research requests, are perceived as barriers to supporting such projects. Within the sector, capturing lived experience data and generating case studies was described as commonplace, paired with an intent to set up specific participation groups across the region to contribute to research. However, to do so, participants expressed the need for research leads and collaborations to clearly articulate how academic and local authority-based studies align to community need and how findings will influence policy and/or practice.

We do have visits from people doing research and they just sign my people up for it. I don’t quite know what happens to them after that. There’s quite a few like national things, researching into dementia at the moment… And I know we work with the hospitals. Oh, the trainee people at UWE and Exeter, we work with them in some respects. – VCFSE organization

There’s no ‘hard’ data… it’s all about the story, and the impact you’ve had on clients, and our ability through a narrative to engage with people that previously we haven’t been able to. – VCFSE organization

Moreover, a major barrier to routinely undertaking more equitable and meaningful participation within the local authority was supporting infrastructure and capacity, with those officers who are trusted by communities often not in a position of power or influence.

My dream version [of research] would be a way of [capturing] knowledge that is much more open and much more, kind of, integrated into people’s lives. I don’t feel like we’ve got the infrastructure to support that at this point… can we create an infrastructure where we trust each other, where we have relationships with each other, where we know how to ask good questions of each other in a curious, compassionate, non-judgmental way and actually a lot of people don’t have any training on how to do that. – Local authority (Public Health)

I think the challenge is often you have people that are trying to do that, at least from my experience, and have got really good skills and are really trusted, but then they don’t have the power behind them to respond to what they’re hearing… there’s a real mismatch between the listening and the action. – Local authority (Public Health)

VCFSE participants suggested an interest in acting as trusted, partner organizations that can support the local authority, and wider organizations seeking to undertake research. Such collaboration could be utilized to actively involve, engage and co-produce research with communities more meaningfully, and embed lived experience in local authority policy-making, service design and decisions.

You’ve got to have somebody that you can trust. And we came to the conclusion, in our group at least, that it needed somebody like me, somebody that’s trusted [by residents] to be approached. – VCFSE organization

### Utilization of data and evidence

Our data highlighted numerous similarities and differences concerning the use of data and evidence within local authority and VCFSE settings. Within the local authority sector, a greater reliance on quantitative data, sourced through routinely collected data, and associated internal datasets were reported as key sources of data and evidence. In contrast to this, our sample of VCFSE participants highlighted lived experience data to be the most sought-after source of data, frequently utilizing surveys and focus groups to gather information. Whilst qualitative data were collected within the local authority setting, our data suggest this to be sparse.

That’s a big piece of work at the moment. We’re calling it the Transform Strategic Insight Report, and that’s using all the data that we’ve pulled in from across our partnership, [and] putting it into our data lake. – Local authority (Children’s Services)

It’s qualitative data that you’re, you’re looking at. It’s not, you know, not hard facts and figures. And you’re talking about experiences for people rather than output necessarily. – VCFSE organization

All participants reported the use of secondary data through national sources such as the Department for Transport, Census, Fingertips, Ofsted, and the Office for National Statistics; alongside locally curated datasets such as Somerset Trends, Somerset Intelligence and bespoke data dashboards, such as the Somerset Insights Hub, specifically developed for, and by, the VCFSE sector. Across both settings, such data were often utilized to inform service and project design, alongside day-to-day decision-making. This enabled staff to understand where the greatest need may lie for certain services or projects, or to provide a rationale for commissioning decisions.

We’ve got a website called Somerset Trends and most of the data that we use for that and for other things is typically Office of National Statistics and government agencies, government departments kind of data that’s published. – Local authority (Economic Development)

However, the inability to either access or share data both within and across organizations was deemed as a considerable challenge, often resulting in the underutilization of available data and duplicated data collection at an organizational and system level. Despite a wealth of routinely collected quantitative data within the local authority, concerns were raised regarding the usability of such data, alongside the applicability of national data to Somerset as a region. To maximize the use of available evidence, local authority staff proposed a supporting infrastructure which enables safe data sharing, in addition to the collection of hyper-local data. Moreover, within both settings, the need for data and evidence to be translated into usable intelligence through training and infrastructure development was recommended.

Sometimes there’s other practical barriers, often around the data, so the wrong data was collected, the data isn’t collected or you can’t collect the data, or you can collect the data but you can’t use it, or we can’t share it and match it with other things. – Local authority (Public Health)

### Research and evaluation training needs

Local authority participants indicated an interest for training in both research and evaluation topics. Specifically, participants expressed a desire for training which supported staff to: develop clear research questions, rigorously collect and analyse qualitative and quantitative data, meaningfully involve community members throughout the research process, and data triangulation. Furthermore, the need for training which enables local authority staff to robustly evaluate services, projects, policies and partnership working was identified. Training in specific evaluation methodologies, such as realist evaluation or ripple effects mapping, was also deemed to be useful for local authority staff.

I think we’re very good at collecting certain types of data, and the Council’s very good at collecting certain types of data. But what I’m not aware of, and sure of, is how we connect these points together to give us a much richer picture of before and after and the impact it’s having on our residents. – Local authority (Transport)

Data further suggest that more general training on broader topics, such as health inequalities, literature searching, data validity/reliability and knowledge mobilization, would further contribute to the upskilling of local authority staff. However, frequency and depth of research and evaluation training must be proportionate to the time, capacity and usage of research within local authorities. Participants recommended that all training should be offered as an introduction to the concepts reported above, with more bespoke training available on a needs basis.

I feel like I’d really like to explore and get more training around inequalities, which in the same breath I feel like I could potentially list them but the examples that we heard and saw this morning were so very real. – Local authority (Local Community Network)

Amongst VCFSE participants, there were mixed views regarding the need for research training, yet overall, its benefit was recognized. Participants demonstrated an interest in introductory training in statistical data analyses and how to more robustly collect and analyse data. Furthermore, an appetite for training around different approaches to engage and collaborate with communities was expressed, alongside support in how to communicate research opportunities. The latter of these training needs was considered to better equip VCFSE organizations with the skills to confidently communicate the value to health and well-being of participating in research, what a project would entail and how their contribution would be used, all of which were acknowledged as a mechanism for improving intersectoral collaboration in research.

Training to tell people what research is about, what is happening, how they can maybe engage [and] participate in that. I’d be interested to learn more about that really. – VCFSE organization

Moreover, the need for specific training in calculating social value was identified and suggested as a tool for VCFSE organizations to clearly articulate the impact of their programmes. Such training was suggested to support future funding generation and commissioning of successful interventions. Furthermore, VCFSE organizations reported a training need for dissemination and communication of evaluation reports or outputs, to share the impact of their work more widely and effectively.

## Discussion

This study provides empirical evidence of how research culture is conceptualized and enacted across local authority and VCFSE sectors, emphasizing the influence that research and evaluation can have on decision-making when applied to the building blocks of health. Utilizing a multi-methods qualitative study design and analysis of focus groups, interviews and surveys identified four key themes. To assess research culture and capacity, HDRCs have typically undertaken surveys, rather than qualitative or mixed-methods approaches [24]. To our knowledge, this is only the second in-depth published account of qualitative findings from an HDRC’s assessment [29].

### Culture and capacity

Our findings suggest that research is highly valued within the local authority, and is frequently used in day-to-day decision-making and in developing policies, strategies and services. Similar findings have been reported in local authority contexts, whereby research is deemed essential to the design and delivery of Council services and interventions [6, 7, 24, 30]. Evidence suggests that engagement in research amongst health and care staff can have a direct, positive effect on the care provided by practitioners [31]. Engagement in research has been reported to lead to the development of research networks, focused on policy and organizational changes to address the so-called time lag between research and practice, strengthened links between academia and communities, practice-focused research, and practitioner skill development – all of which were reported to improve the offer of care, reflecting the value of engaging in research [31]. However, a majority of literature Boaz and colleagues (2024) draw upon is positioned within clinical healthcare, and predominantly cancer care. Whilst still in its infancy, HDRCs and funders need to ensure that both positive and negative impacts of research engagement in local authorities is captured to inform future commissioning and practice.

However, to understand the impact of research engagement within local authorities, a shared understanding of research in this setting is required. Both within our findings and the wider literature, research as a term in this setting is contested and is typically considered the application of evidence or data, as opposed to the collection and analysis of primary data [6, 24, 29]. Differences in how research is defined across settings risks local authority staff perceiving that they are not research-active, despite actively using evidence and data within their roles [30]. The lack of a consensus definition that reflects the specific research activities undertaken in local authorities has been claimed to act as a barrier to the development of research culture both within individual organizations and the sector [32, 33]. Acknowledging the difference in the definition of research between local authority and academic or clinical settings is vital to ensuring that evidence and data are applied in decision-making within Councils. This has recently been supported through the development of a consensus definition for research in local authorities:

Part 1

Local authority research supports decision making about practice, policies and interventions at a local, regional or national level

and/or

It helps us understand how people are impacted by the context in which they live, work and go about their daily lives.

AND

Part 2

Research uses structured, organised and reproducible methods to:

produce new information or knowledge, which may include testing an idea, theory or new intervention

and/or

provide a new interpretation of existing information. This may include routinely collected data being used for a new purpose, as well as publicly available data. [33, 34].

The consensus definition demonstrates the need to broaden how research is defined within local authority, giving equal focus on collecting new data and utilizing routinely collected data beyond performance monitoring. Whilst there are similarities in definitions of research adopted in other settings, such as the NHS or academia, a consensus definition for local authorities enables staff to identify where they are already, or could become, involved in research [33]. Subsequently, more local authorities may begin to consider themselves as research-active given the context-specific definition. The usefulness of the definition is yet to be determined, and persistent challenges such as gaining ethical approval for research are likely to remain [32, 33, 35]; however, the definition offers clarity and enables better communication around research within local authorities, contributing towards the development of research cultures for evidence-informed decision-making [33]. Importantly, this definition recognizes the need for structured, organized and reproducible methods, which promotes rigour, reliability and validity within local authority-based research. However, to do so, it is recommended that not only bespoke research training is required to better enable staff to undertake research but also organizational culture and system change [6, 30]. Our findings report an interest and need for local authority staff to have training in a wide range of research and evaluation areas, ranging from research methods to evaluation approaches, and meaningfully involving communities in research; yet our findings also indicate the need for infrastructure and system change whereby governance processes, easier data sharing procedures, access to research, and protected time and capacity for research and evaluation are enabled. Through HDRCs, and other local authority research promotion schemes, such as the Research Practitioner scheme, addressing such barriers would better position local authorities to collect and utilize primary data, resulting in more bespoke or refined strategies, policies and services that meet communities’ needs. Further research which explores local authorities as complex systems and identifies the key leverage points for developing research culture and capacity is required.

### Collaboration

Our findings indicate that there is a need to develop how lived experience could be better integrated into decision-making within the local authority, focusing on meaningful involvement and equal partnership. Predominantly, existing community involvement has been actioned through consultation and feedback mechanisms, a commonly utilized approach by local authorities [7]. Whilst such an approach does enable community voice to contribute towards local authority decision-making, participants within our study demonstrated a desire to shift from consultation and feedback to more community-participatory approaches. In efforts to address this challenge, Somerset HDRC has recently implemented a lived experience advisory panel to support research on health-related economic inactivity, with an evaluation of this activity underway. Establishing such partnerships is essential to undertake impactful research with communities and therefore vital in understanding the complexity of people’s lives, and their health needs and priorities [36, 37]. Community participation infrastructures are highly recommended mechanisms for developing trust with communities and co-creating actionable outcomes for research, practice and policy [36, 37].

Our results suggest that fostering equitable research partnerships between the local authority and VCFSE organizations through harnessing: (i) local authority’s recognized need to better involve communities in decision-making; (ii) local authority’s need to develop greater trust with communities; (iii) VCFSE organization’s expertise in working with communities and collecting lived experience data; (iv) VCFSE organization’s interest in establishing community research groups; and (v) VCFSE organization’s desire to be involved in local research, may prove an effective mechanism for integrating community voice into decision-making within the local authority. However, to effectively undertake this, our findings suggest that clear pathways to impact, which outlines how lived experience data will be used to inform practice and policy, accompanied by dissemination of research findings to community members and VCFSE organizations, is required.

Moreover, a major concern of community involvement in research within the United Kingdom has been navigation of the power dynamic between researchers and communities [38]. Community-based participatory research (CBPR) approaches offer an effective way of creating equitable partnerships with community members and organizations, whilst simultaneously negating power dynamic concerns (if carried out correctly) and generating local data to inform policy-making and local public health priorities [36, 39, 40]. Whilst CBPR approaches offer an opportunity to build sustainable co-production processes and provide mechanisms to involve communities in research from start to finish, appropriate remuneration is essential, which may prove challenging in times of financial difficulties within local authorities. In turn, local authorities may wish to consider alternative and tailored approaches to reimbursing participants such as access to training or opportunities for skill development.

In light of our findings and given the substantial growth in financial backing of local authority-based research within the United Kingdom, whereby 30 HDRCs in the country have been formed, amounting to ~150 million pounds sterling of government funding between 2023 and 2029 [41], it appears that there is a significant opportunity to reform how lived experience data are used to inform local authority decision-making. Through local authority research infrastructures, further exploration of how CBPR principles can be adopted to inform research, policy and practice centred on the building blocks of health is required. Specifically, future research should not only explore the acceptability and effectiveness of such an approach to community participation but also equally seek to understand how CBPR approaches and local authority–VCFSE partnerships could be sustainably integrated into local authority research culture and practices.

### Strengths and limitations

This study builds upon the limited reporting of HDRCs’ baseline assessments of research culture and capacity within local authorities in the United Kingdom, offering insights which span beyond public health and explore the views of those within services such as housing, education, economic development, and children’s and adult services. Senior leadership perspectives were not captured owing to the pressures and restructuring of the local authority at the time of data collection, therefore limiting the strategic insight within our data. Moreover, internal challenges such as these may have further limited sample size due to job uncertainty. Additionally, despite multiple contact attempts with a range of organizations, focus groups, interviews and qualitative surveys offered as alternative forms of data collection, our VCFSE sample was small. Whilst this limits the generalizability of our findings for the sector, this challenge represents the importance of language and communication around research within different sectors. Future studies seeking to better understand research within the VCFSE sector should consider use of language with focus on concepts including evaluation, service design and service improvement. Our multi-methods approach gathered qualitative data and therefore, whilst our sample size may be smaller than other HDRC studies, which utilized quantitative approaches, our results offer an in-depth analysis of research culture and capacity within the local authority. Moreover, our research similarly explores research culture and capacity amongst VCFSE organizations, identifying avenues for future collaboration to better integrate lived experience into local authority decision-making. The risk of bias within the collection and interpretation of findings should also be considered. The research team consists of individuals affiliated with a range of organizations, including the local authority from which data were collected. In most focus groups, lead facilitators were not known or familiar to participants, as they either worked in a different department of a large organization or were a non-local authority member of the research team or a newly appointed member of the research team. However, where, in particular some Public Health focus groups involved participants with prior relationships with the researchers, this may have increased the risk of social desirability bias. Whilst this risk cannot be truly negated, the co-facilitators of focus groups were unfamiliar with participants and held responsibility for asking probing questions, a standardized topic guide informed by an appreciative inquiry exercise was utilized (Supplementary Material 1), and neither lead facilitator analysed the obtained data from these focus groups. Additionally, member checking (returning transcripts to participants for verification) was not undertaken, which may limit the credibility and confirmability of the findings. Lastly, it should be acknowledged that this research explored a unitary authority located in a rural and coastal region of England. Therefore, our findings may not be generalizable to other local authorities with different operating structures or located in other parts of the country with different health needs. Despite this, our study offers a novel contribution to the field using qualitative methods and provides tangible recommendations for other local authority research infrastructures to explore within their localities.

## Conclusions

Local councils in the United Kingdom are well-placed organizations for research focusing on the building blocks of health, such as transport, education or planning, and have had significant investment over recent years in efforts to build research culture and capacity. However, to effectively and sustainably address this, an in-depth understanding of existing research practices, interests and needs is required. Data gathered from our multiple qualitative methods approach firstly demonstrate how research in local authority and VCFSE settings differs from research in traditional academic settings.

Our findings not only highlight the complexity and nuances of enabling research to take place in local authorities based solely upon definition but also equally identify more tangible solutions to organizational and system barriers to engaging and undertaking research. Namely, the development of governance policies and processes, internal and external data-sharing agreements, access to research and licenses, protected time for research, and the identification of organizational and system leverage points should be prioritized. Moreover, integrating lived experience into service design, policy and practice was unanimously identified as a priority across both settings. Whilst VCFSE organizations’ expertise, and the local authority’s interests, offer a strong starting point to action this, further practice-based research that explores the implementation, acceptability, effectiveness and sustainability of community participation infrastructures is required.

## Supplementary Information

Supplementary Material 1.

Supplementary Material 2.

Supplementary Material 3.

## Data Availability

The anonymized dataset has been held in accordance with the Data Protection Act 2018 and General Data Protection Regulations on internal Somerset Council software and is not publicly available. Data can be requested via the corresponding author.

## Abbreviations

UK: United Kingdom
VCFSE: Voluntary, Community, Faith and Social Enterprise
NIHR: National Institute for Health and Care Research
HDRC: Health Determinants Research Collaborations
UWE: University of the West of England, Bristol
COREQ: Consolidated Criteria for Reporting Qualitative Research
CBPR: Community-based participatory research
