# Supplementary material for: Understanding existing research culture and capacity in a local authority (LA) and voluntary, community, faith and social enterprise(VCFSE) in one area of the South West, United Kingdom: a qualitative baseline study
Source: Health Res Policy Syst. 2026 Aug 10;24:96. doi: 10.1186/s12961-026-01498-x (PMC13741450; doi:10.1186/s12961-026-01498-x)
Supplement: Supplementary file 2 [file 12961_2026_1498_MOESM2_ESM.docx]

Interview/Focus Group Topic Guide

**Secction 1:**

1) Research often means different things to different people, the Somerset HDRC team are considering adopting the Cambridge Dictionary definition of research: ‘*a detailed study of a subject in order to discover information or achieve a new understanding of it*’ – Is there anything you would add, take away, or change within this definition?

– There are alternative definitions, such as the Health Research Authority definition: *research is defined as the attempt to derive generalisable or transferable new knowledge to answer or refine relevant questions with scientifically sound methods* (which excludes practice audits/service evaluations).

2) How important do you feel is the use of evidence and research in your role? How could research and/or evidence be used in your role?
3) Thinking about the day-to-day activities in your role, what, if any, types/sorts of information/research evidence do you use in your day-to-day activities?

**Prompt/examples**: Are there any specific sources of information/research evidence you use? (e.g National, Regional and Local Data Sources (eg. Fingertips, ONS data, Explore Education Statistics, Housing Survey), National Guidance (eg. CMO reports, NICE guidelines), Research journals, NIHR Evidence Summaries, Internal data sources (eg. Public Health Intelligence, JSNA, Service level datasets))

**Section 2:**

4) What are your thoughts/views on the usage of research and evidence in, or by, your service/directorate?

**Prompts:**  Is the use of research and evidence promoted within your service/directorate? Can you give an example of how R&E has been promoted? Have you ever conducted an economic evaluation on any of your services? If yes, who carried this out?

**Examples:** Promotes evidence from new research; promotes evidence-based practice; Senior managers who support research, working with external, easy access to journals and research articles, encourages engagement with research-related activities, has identified experts, partnering with academic institutions, or support, for research, promotes staff career development/pathways in research, has a policy/strategy for research

5) If your service/directorate promotes the use of research, to what extent, if at all, are community groups and/or members involved in the work?

**Prompts:** How are, or could, they be involved in the research process? Are there any successful/unsuccessful examples of this?

6) What, if anything, enables and encourages you in using evidence and research within your day-to-day work?
7) What are the barriers, if any, to you in using evidence and research within your day-to-day work?
8) Have you ever had the opportunity to be involved in research, either as part of the research team, or as a research participant, during your current role? If yes, please could you tell us more about this opportunity?

**Prompt:** How did the opportunity come about? Who was it with? In what capacity were you involved?

9) Would you like to be (more) involved in research in the future? If yes, how do you think this could be best facilitated? If no, could you please explain your reason/s?

**Section 3:**

10) Have you previously taken part in any research training?

- If so, what were they? Who were they delivered by?
- If not, are there any specific reasons why you haven’t engaged in any research training?

11) In what area(s) of research and/or evidence do you feel training would be most useful or interesting to you and/or your service?

- **Examples:** Quant and qual, stats, critical appraisal, literature searching & reviews, community participatory research, co-production, grant writing, research ethics, research design/proposals

11a) In what area(s) of evaluation do you feel training would be most useful or interesting to you and/or your service?

- Examples: economic evaluation, process evaluation, ripple effects mapping, logic models/theory of change

12) What would support/facilitate you to engage in future training in these topics?

- **Prompt:** What, if any, type of support would you need? Are there any specific barriers to you taking part in research training? How could these be overcome?

13) What would be your preference in how training is delivered?

- **Examples:** Online webinars/workshops, in-person seminars/workshops, combination of both

**Section 4:**

14) Within your role or team/directorate, are you aware of any research opportunities to inform/strengthen evidence-based decision-making?
15) Reflecting on the work you do, have you identified any gaps in evidence and research that could improve service design or delivery?
16) Thinking about your area of practice, how do you see inequalities linked to factors such as income, rurality, homelessness, gender, ethnicity, or other protected characteristics, affecting your *customers/communities*?

(Examples to be given using local datasets available within Somerset Council)

**Prompt:** Of these factors, which do you think are the most prominent in their contribution towards inequalities in your area of practice?

17) Thinking about the above, what evidence, data or information do you believe could help us to reduce some of these inequalities?

**Prompt:** Do you have any ideas about how we might be able to gather or collate such evidence and data?

- what expertise and/or data and intelligence within your team could be helpful in generating evidence that is useful beyond Somerset?
- **If none**, what are the barriers to generating such data? / If no, how do you think you could be best supported to generate evidence that might be relevant beyond Somerset?
