# Supplementary material for: Understanding existing research culture and capacity in a local authority (LA) and voluntary, community, faith and social enterprise(VCFSE) in one area of the South West, United Kingdom: a qualitative baseline study
Source: Health Res Policy Syst. 2026 Aug 10;24:96. doi: 10.1186/s12961-026-01498-x (PMC13741450; doi:10.1186/s12961-026-01498-x)
Supplement: Supplementary file 3 [file 12961_2026_1498_MOESM3_ESM.docx]

| **Theme** | **Subtheme** | **Code** | **Code Definition** |
| --- | --- | --- | --- |
| 1. **PERCEPTIONS AND VALUE OF RESEARCH** |  | |  |
|  | 1.1 What Does 'Research' Mean | i. *finding out/collecting information* ii. *complex academic work* iii. *desk-based research* iv. *using existing data* | i. Collecting, gathering or finding new or existing specific information relevant to a chosen topic ii. Research is undertaken by universities, and involves complicated language and processes iii. Research can be searching for publicly accessible information online iv. Research is the process of finding and making use of data that has been collected, summarised and made available by other organisations / individuals |
|  | 1.2 Research Activity | i. *desire to get more involved in research/evaluation:* ii. *multiagency / intersectoral research collaboration* iii. *‘we don’t do research’* iv. *examples of research* | i. Interest in undertaking, leading or collaborating on research and/or evaluation projects ii. Working in partnership with other organisations to undertake research/evaluation iii. Research is not considered as the responsibility or remit or a team or individual iv. Specific examples of research activities |
|  | 1.3 Research Language and Communication | i. *messaging around research (information and opportunities)* ii. *accessibility / inclusive language* iii. *feedback of participation/results* | i. How research studies, findings, and opportunities are discussed, promoted, or shared ii. How accessible, inclusive, and/or lay language is used to talk about research iii. If, and how, research study findings are shared back to VCFSE community groups and residents |
|  | 1.4 How is Research Used | i. *decision making and service improvement* ii. *efficiency and cost-saving* iii. *annual reporting* iv. *funding applications* v. *strategic influence* vi. *creating / sharing best practices* | i. Research is used to inform how policy, service design, or project development decisions are made ii. Using research to find more efficient ways of working, either creating or striving for cost savings iii. Research as another term for collecting information about a service, project, or organisation yearly performance iv. Undertaking or using research to support funding or grant applications v. Using research to convey messages to senior leaders, and/or within strategic decisions vi. Using research to develop and share examples of effective ways of working |
|  | 1.5 What Does Evaluation Mean | i. *getting feedback about an action* ii. *information for change* | i. Understanding what did and did not work within a service ii. Gathering information and insights to refine, adapt, develop or improve existing or new services or projects |
|  | 1.6 Barriers to Research | i. *time and capacity* ii. *lack of supporting resource and infrastructure* iii. *lack of funding* iv. *survey fatigue* | i. Not enough time within day-to-day roles and responsibilities to engage with research ii. No or limited access to research resources, specialists, or training iii. No or limited funding allocated to research engagement/activity iv. Overdistribution of surveys across the system reduces desire to complete research surveys |
| 2. **COMMUNITY PARTICIPATION** |  | |  |
|  | 2.1 Attitudes and Perceptions | i. desire to do more | i. Interest and willingness to work more with communities at any point(s) throughout the research/evaluation process |
|  | 2.2 Existing Practice/Infrastructures | i. *types of community participation undertaken* ii. *consulted* iii. *involved as research participants* iv. *not regarded as co-researchers* | i. Examples of community participation activities in research/evaluation ii. Examples of communities being consulted on proposals, research plans iii. Examples of involving residents as participants in research studies or evaluations iv. Residents are not seen as equitable members or a research project and/or team |
|  | 2.3 Challenges in Undertaking or Participating | i. *hearing from the right people* ii. *community awareness of research* iii. *community trust* iv. *co-production* | i. Difficulty in integrating diverse and representative voices into research, evaluation, and routine practice ii. Communities are not familiar with research processes or purposes iii. There is a lack of trust in public/statutory bodies to improve services for communities iv. Inappropriate use of co-production as a term |
| 3. DATA AND EVIDENCE |  | |  |
|  | 3.1 Research Methods Used | i. *community-responsive* ii. *quantitative* iii. *qualitative* iv. *mixed-methods* v. *community voice/lived experience* | i. Community-needs/interests inform the research method used ii. Examples of quantitative research methods used iii. Examples of qualitative research methods used iv. Examples of mixed-methods research methods used v. Examples of gathering lived experience of residents and service users as a research method |
|  | 3.2 Sources of Data and Evidence | i. *service user / community feedback* ii. *national datasets* iii. *internal datasets* iv. *knowledge transfer* v. *best practice* | i. Examples of service user feedback and/or lived experience as a source of data to support decision making ii. Examples of national datasets used as data to support decision making iii. Examples of routinely collected data and/or internally developed datasets to support decision making iv. Examples of professional knowledge used to support decision making v. Examples of using professional or regulatory bodies’ guidance to support decision making |
|  | 3.3 Barriers to Using Data and Evidence | i. *data sharing* ii. *poor data quality* iii. *knowing how to use routinely collected data* iv. *time, capacity & resource* v. *lack of local data* | i. Difficulties or inability to share data either internally or externally limiting the use of data for decision making ii. Missing, incomplete, or unreliable data limiting the useability of data iii. Limited knowledge or skills in using routinely collected data beyond statutory reporting requirements iv. A lack of time, competing priorities/workloads, funding, personnel, and/or organisational pressures acting as a barrier to utilising data and evidence for decision making |
| 4. **TRAINING, ASSETS, AND SUPPORT NEEDS** |  | |  |
|  | 4.1 Appetite for Training |  |  |
|  | 4.2 Training Needs | i. *research methods* ii. *using routine data* iii. *project/policy evaluation* iv. *report writing* v. *self-directed training* vi. *upskilling* | i. An expressed need or interest in training on general or specific research and/or evaluation methods ii. An expressed need or interest in training focused on how to best utilise routinely collected data or information iii. An expressed need or interest in training for evaluating projects and/or policies iv. An expressed need or interest in training on how to write up research or evaluation projects v. An expressed need or interest for external, self-directed training opportunities in research/evaluation vi. An expressed need or interest in general research skills or fundamental skills (e.g. ethics, critical appraisal) |
|  | 4.3 Infrastructure Needs | i. *literature access* ii. *central research/analysis team* iii. *funding as a facilitator of research* | i. An expressed need or interest in receiving support to access published, peer-reviewed research ii. An expressed need or interest in receiving support from a specialist research team within the Council iii. The need for funding to enable research to take place |
|  | 4.4 Culture Needs | i. *senior management buy-in* ii. *integrating research as business as usual* | i. Senior leader’s approval, promotion or adoption of research as an activity is a direct facilitator building research culture ii. Research and evaluation must become part of everyday practice to achieve culture change |
